# Community- Based Approaches to Cancer Prevention in Rural Thailand Based on Experiences of Accredited Health Professionals

**DOI:** 10.31557/APJCP.2020.21.1.7

**Published:** 2020

**Authors:** Kesinee Saranrittichai, Wichai Ussavapark, Apisit Thamrongwarangkoon, Tiptiya Heangsorn, Sureerat Daoruang, Ampornpan Teeranut

**Affiliations:** 1 *Research and Training Center for Enhancing Quality of Life of Working Age People, *; 2 *Faculty of Public Health, *; 6 *Faculty of Nursing, Khon Kaen University, *; 3 *Director of Nampong Hospital, *; 4 *Director of Ubolratana Hopital, *; 5 *Professional Nurse, Nampong Hospital, Khon Kaen, Thailand. *

**Keywords:** Cancer prevention, community, health service skill, health literate community

## Abstract

This qualitative research aimed to describe important components of community based approaches of cancer prevention from working experiences of accredited health professionals in Thai rural communities.

**Methods:**

Data were collected from 23 communities with 23 accredited health professionals as key participants using in-depth interview and focus group discussions (FGDs) in each community. Other informants were community leaders, community health volunteers and community people. Content analysis was applied for data analysis.

**Results:**

Community based approaches for cancer prevention from the experiences of accredited health professionals were composed of 2 themes: approaches for community-based cancer prevention and methods for cancer-based prevention in the community. The approaches for community-based cancer prevention was composed of 4 components: 1) primary prevention focusing on health education; 2) secondary prevention include: life style modification, vaccination, advocacy of cancer screening, early detection, and prompt refer; 3) tertiary prevention focusing on primary care, and 4) research collaboration focusing on net working. Methods for cancer-based prevention in the community were different based on types of cancer. The 3 factors determined to be associated with community based approaches for cancer prevention were: 1) primary care policy; 2) hometown workplace; and 3) health practice skill.

**Conclusion:**

Community based approaches for cancer prevention should concentrate on building a literate community involvement to empower both health care providers and residents. From our evidence, dedicated community associates health professionals have a major role to play.

## Introduction

Cancer is one of leading causes of health problems worldwide, with lung cancer, breast cancer and prostate cancer being particularly important (Bray et al., 2018). Cancer prevalence and incidence increases until ages 85–89 and overall cases have increased over time (Nolen, 2017). It was found in Thailand that cancer-related deaths are due to risk behaviors and environment such as diet, tobacco, infection, radiation, stress, physical activity, and agents such as pollutants (Anand et al., 2008). Most common risk factors of cancer are smoking (55%), diet (20%), genetics (20%), alcohol drinking (19%), and other lifestyle components (17%) (Marlow et al., 2012), as well as aging due to older tissue proneness to the action of environmental carcinogens (Balducci, 2016). Previous study showed older person had higher prevalence of positive Fecal Immunochemical Test (FIT) than other group (Sarakarn et al., 2017).

Lack of physical activity makes a contribution (Hoffman, 2016). People with cancer are faced with multiple problems such as physical vulnerability, cognitive decline, co-morbidity, altered social contexts and lack of life perspectives (Ebenau et al., 2017). 

National policies and programs should be focused on prevention by raising awareness, reducing exposure to cancer risk factors and providing information and support to adopt healthy lifestyles. People who have awareness of actual cancer causes were more likely not to smoke and eat sufficient fruit and vegetables (Shahab et al., 2018). Approaches need to be focused on both healthy individuals and high cancer risk groups (Albini et al., 2016). It was found that smoking cessation up to age 70 was associated with a significant decrease in the incidence of lung cancer (Balducci, 2016).

Cancer morbidity and mortality can also be reduced through screening and prevention (Angier, 2017) basically divided into: (1) primary prevention, which aims to prevent the onset of disease by altering cancer risk behavior (Gordon, 1983), with approaches to build environments reducing exposure to risk factors (Gomez et al., 2015) and healthy lifestyles promotion (Balducci, 2016); (2) secondary prevention, which encompasses screening and early diagnosis to slow or stop cancer progression (Gordon, 1983), and thus reduce the morbidity and mortality associated with cancer (Balducci, 2016), build special environment for healthcare and cancer screening resources in a community (Wray, 2018); and (3) tertiary prevention, which aims to prevent recurrence or progression of disease by medical interventions (i.e., chemotherapy, radiation treatment, and surgery) (Gordon, 1983) and reduce further complications, disabilities or suffering. People with cancer need to care and attention to quality of life, in particular at the end of their lives. 

A previous study focused on the 4Es: enabling, empowering, enhancing, and evolving effects on mental well-being of people with cancer and their contributions to quality of life (Chan et al., 2018). Home is a place of achievement, triumph. comfort, belonging, social connection and collaborative caring of people (Horsfall et al., 2017). People with cancer often view support in terms of their abilities to spend their lives in their own ways at home (Maersk, 2018). Cancer prevention needs to be implemented at community level. Approaches need to be focused on establishing working partnerships with community leaders and stakeholder organizations (Weiner and MacDonald, 2013). 

Since cancer prevention needs to be family-, community-, and hospital-based, multi-disciplinary teams including epidemiologists, and public health specialists, nurses and doctors are needed to optimize the chance of success (Saranrittichai et al., 2012). Accredited health professionals can play a major role in community based care. Included are a special group of public health officers and nurses who work in urban or rural hospitals but additionally volunteer to take care of people in their own rural communities. The benefits of their participation can be seen in increasing capacity for action and acting as role models to improve cancer prevention efforts in their own communities. 

## Materials and Methods

This qualitative study aimed to explain important components of community based-approaches for cancer prevention based on accredited health professionals’ work experience in rural area in Khon Kaen province, Thailand. 

Study sites were 23 communities and the key informants were a total of 23 accredited health professionals currently working in rural or urban hospitals but volunteering to assist in their home town or rural communities for at least 3 years. They set themselves the tasks: 1) good Buddhist practice; 2) reduce risk behavior such as smoking, and alcohol consumption in at least 10 community people; 3) take part in health promotion, health prevention, caring and rehabilitation in their own communities; 4) apply the Self - Sufficiency Economy (His Majesty King Bhumibol Adulyadej’ s theory) in community people ways’ of living; and 6) be accepted and chosen by community people as health professionals close to their hearts. 

Data were collected by: 1) two in-depth interviews with 23 accredited health professionals individually, each taking about 1-2 hours at their work places; 2) focus group discussions (FDGs) performed once in each community separately. Participants in each FGD were 12-15 representative community leaders, health volunteers, and community people. Each FGDS was about 60-90 minutes in duration. Data collection was continued until data was saturated. Content analysis was done simultaneously with data collection. 

## Results

Results were presented in 3 parts; 1) general information, 2) community-based approaches for cancer prevention from the experiences of accredited health professionals and 3) factors associated with community based approaches for cancer prevention

*General information*

The total of 23 accredited health professionals were mostly female. Most of them were aged 46-50 years, were married and had graduated with a master degree in nursing. They had experience of working for more than 16 years, taking a variety of training courses in different areas such as nursing practice, health care service, advances practitioner skills, health policy, health system, health care reform, community based care, and the self-sufficiency economy theory application.

*Community-based approaches for cancer prevention from the experiences of accredited health professionals*

It was found that community-based approaches for cancer prevention from the experiences of accredited health professionals was composed of 2 themes: 1) approaches for community-based cancer prevention; and 2) methods for cancer-based prevention in the community.

*Approaches for community-based cancer prevention*

It was found that community-based approaches for cancer prevention were conducted to ensure participation of community people. As the accredited health care professionals said “What we have done, we need to get people involved and make them aware of their health problems and causes. They also need to find out their own ways to solve their own health problems. They need to help themselves”. The community-based approach was composed of 4 components: 1) primary prevention focusing on health education in 3E 2S principle: exercise, eating, emotional, smoking, and alcohol (Alcohol means sura or loa in Thai); 2) secondary prevention include: life style modification in risk group by using 3E 2S principle, vaccination, advocacy of cancer screening, early detection, and prompt refer; 3) tertiary prevention focusing on prompt primary care; and 4) research collaboration focusing on networking. 

Based on Thai health policy, the 3E 2S principle was generally implemented for health promotion and health prevention and integrated with way of living of community people. Thus, 3E 2S principle were applied for cancer prevention in all primary, secondary and tertiary prevention but stricter in risk groups. More details are shown in Table 1.

*Methods for cancer-based prevention in the community*

Different methods were used in different types of cancer in nature: liver cancer, cholangio carcinoma, lung cancer, colorectal cancer, cervical cancer and breast cancer. Interestingly, it was found that accredited health care professionals always done situational analysis by exploring problems and needs of community people before implementing any methods for cancer-based prevention under their own contexts. This could provide the easy and practical way for community people to conduct themselves for cancer prevention. More details are shown in Table 2. 

*Factors associated with community based approaches for cancer prevention*

The 3 factors determined to be associated with community based approaches for cancer prevention were: 1) primary care policy; 2) hometown workplace; and 3) health practice skill. 

*Primary care policy*

Primary care policy, encouraging and supporting accredited health professionals to work in their own communities, contributes accredited health professionals to have the opportunity to work for their home villages. Some accredited health professionals said “Thank you for the hospital policy. It provides us the chance to work for our villages”. In addition, primary care policy include local policy set up by communities to solve their health problems. One community leader said “We have the policy for quit smoking, everyone who would like to quit smoking need to sign contact with the community”. One health volunteer said “We need to follow our commitment that our community will not have any alcohol on funeral ceremony.

*Home town workplace*

Working in hometown help accredited health professionals to understand community people and their ways of living. A sense of joy and happiness from working in home town makes them feel that they are valuable that they can change people’s lives and help others. This is the inspiration of the accredited health professionals allowing them to happily continue working. One accredited health professional said “It’s easy and convenient for me to work. I help my relatives or my own community people. I am so happy when I see them getting better”. Another one accredited health professional said “I didn’t do something much, only talk to the family members and give them small support on my way home. I finally found that her life is changed, her cancer wound is healed and she can help her community. This is one example of inspiring me to work in the community”. 

*Health practice skill*

As all accredited health professionals were knowledgeable and experienced working for more than 16 years. They also had been trained in many courses regarding health practice such as health promotion, health prevention, health care practice, cancer screening and multidisciplinary team working, which can be integrated and contribute to effective cancer prevention in the community. One accredited health professional said “I was trained to do per vagina (PV), Thus, I do pap smear for CA cervix screening”.

**Table 1:** Approaches for Community-Based Cancer Prevention

Themes and codes	Approaches and illustrative quotes
Primary prevention	Primary prevention focused on education.
Education	Education was to set up training course and got community people involved in training course for health promotion and health prevention. One accredited health professionals said *“We set up training course for community people to access to information, understand, make decision and use information for self-management to prevent cancer based on 3E 2S principle which are exercise, eating, emotion, smoking,, and alcohol”.*
Secondary prevention	Secondary prevention focused on life style modification to reduce risk behaviors with regards of 3E 2S principle, vaccination, and advocacy of cancer screening, early detection, and prompt refer.
Life style modification	Life style modification was based on 3E 2S principle, in particular with risk group. Before implementing life style modification, accredited health professionals always explore people at risks problems and need. One accredited health professional said *“We ask community people to draw their goals, how can they meet their goals, make people aware of their health problems by getting them realize and involve in cancer prevention, plan, making decision and solve their problems by themselves. We are their facilitators”. *Another one accredited health professional said* “We asked them to find out what are their health problems? What are these causes of problems and how can we solve these problems under their own contexts?”*
*Exercise*	Exercise focused on explore problems and need of exercise of community people. Then get them decide when, where, and how to exercise in different groups of people in the community. One accredited health professionals said *“For elderly people, they would like to do aerobic but for adolescent, they would like to dance and play sports. We set up exercise group to do aerobic in the evening 3 days a week. But for adolescent, they set up their own group to do dancing after school or on weekends”*. One community member said *“My son and his friends play football. They also participate in football competition in the community. Our accredited health care professional is one of our committee to support this activity”.*
*Eating*	Eating means eat clean food and not contaminate with carcinogen. One accredited health professional said “*We have the project “grow what we eat and eat what we grow”*. Community people growing plants and sell crops from their farm. They use less and less chemical substances less and less. One community member said *“This means that we grow and eat organic vegetable and fruit. People eat clean food, organic vegetable and fruit more and more’. *
*Emotion*	Accredited health professionals said *“We developed training course for community people to decrease stress. They have to consider what exactly their health problems and causes. They also need to solve the problems by themselves. We are their facilitators. We found that they their families were happy when they could solve their own problems. We also found that they had better health”*. One community people said *“Since I have applied the Self - Sufficiency Economy theory into our ways’ of living. We grow what we eat and eat what we grow. We sell our crops. We have more money and no dept. My son went back home to help our family. Amazing! I used to take medicine to control my blood pressure, but now I don’t”. *
*Smoking*	Smoking was focused on commitment and commitment. One accredited health professionals said *“We set up self-help group in the community for them to help each other to decrease smoking. We found ones who were success with quit smoking and they volunteered to be role model for quit smoking in the community. I empower them”.* One male community member said *“Since I have the commitment with my accredited health care professional to quit smoking, try to do it and finally I can quit smoking for 8 month”.* One community leader said *“We have found that our community member can quit smoking both adolescent and adult”. *
*Alcohol *	Alcohol reduction focused on local policy implementation. Accredited health care professionals support community policy for alcohol consuming reduction on a merit making ceremony in particular “Ngan Sop Plod Loa”. (means “No alcohol in funeral ceremony”. Ngan means ceremony. Sop means funeral and Loa means alcohol). One community said *“I don’t have any alcohol on my father’s funeral ceremony. People didn’t ask for alcohol. I save my money a lot”*. One health care volunteer said *“Our accredited health professional educate us on alcohol consumption and support us to reduce drinking alcohol. Now we don’t have any alcohol on funeral ceremony. We can change our lifestyle with this policy”.*
Vaccination	Vaccination focused on providing vaccination according to Thailand health policy, National Cancer Control Programme. Accredited health professionals said *“We do vaccination for cancer prevention with children and people who are at risk and prone to have infection contributing to cancer according to Thailand health policy such as hepatitis B vaccine. For Human Papilloma Virus vaccine, we now provide for all female student who study in primary school the age of 9-10 years”. *
Advocacy of cancer screening, early detection, and prompt refer	Community people said *“We participate in cancer screening in our community, accredited health professional teach us about breast self-examination”*. One accredited health professionals said *“We do cancer screening for breast cancer and colorectal cancer as we have high incidence rate of these 2 types of cancer”*. Another one said *“When we do cancer screening and found something’s wrong, we need to do early detection and prompt refer patient to have proper treatment. We also educate and encourage female the age of 30-60 years old to do breast self-examination”*. One accredited health volunteer said “*We encourage community people both male and female the age of 50-70 involved in colorectal cancer screening using FIT test (Fecal Immunochemical Test).* Another one said *“If we found something wrong, will promptly refer the patient to see the doctor”.*
Tertiary prevention	Tertiary prevention was to prevent complications of cancer in patients with cancer and do prompt primary care. One accredited health care professional said *“Health volunteer help me to find out the patient and do home visit. I visit a woman with cancer at home, she has cancer wound on her left breast, It’s a big open wound and smell bad. She cannot participate with other people. I clean her wound every day and encourage her to eat more vitamin C in oranges and herbs to help wound healing. *One community people said *“My wound was better and better within 2 weeks, there was no smell at all. I can continue my work and help community”. *
Research collaboration	Research collaboration helped easily access to cancer screening. One accredited health professionals said “*We participate in research activity and collaboration with Asean Cancer Epidemiology and Prevention Research Group (ACEP), Khon Kaen University, Thailand. They have colorectal cancer screening project in the community. We get community people involve in FIT”.*

**Table 2:** Methods for Cancer-Based Prevention in the Community

Type of cancer	Methods and illustrative Quotes
Liver cancer	Important methods for liver cancer prevention were Hepatitis B vaccine and decrease carcinogen consumption.
	One accredited health care professionals said *“We give Hepatitis B vaccine to children the age of 1-6 month and for risk group under 19 years old according to doctor’s prescription”*. Another one said *“We develop the project to decrease alcohol consumption in the community. We empower the community to set up local policy for community people not to drink alcohol in traditional festival”.* One community leader said “*We set up project to decrease carcinogen such grilled meat, grilled pork, and grilled chicken, especially alpha toxin in bean or nut. Our health professional help us as our facilitator”.*
Cholangio carcinoma	Important methods for cholangio carcinoma were decrease eating raw fishes and raw fish products in particular with children to prevent liver fluke infection. One accredited health care professional said *“We found that young generation eat raw fish more than previous time. We take part in collaborative projects with government organizations and learning institutes to decrease eating raw fish, fermented fish, and raw fish production with students in primary and secondary school. We educate them and make them aware of cholangio CA, causes, consequences and how to prevent themselves from cholangio CA. We have found that there are some projects implemented by using social media “Sticker Line” for children. They have fun and try to change their risk behaviors not to eat raw fish and decrease eating fermented fish”.*
Lung cancer	Method for lung cancer prevention was empower community people to decrease smoking in particular with children and adolescents. One accredited health professional said *“We found that overall smoking rate is decrease, but increase smoking in young people. What we have done with young smoker to quit smoking is finding out the adolescent leaders to be change agent for quit smoking. We found one who is leader of adolescent gang. We convinced and encourage him to be security man for the community. He did a good job on doing something good for community such as decrease adolescent risk behavior, in particular quit smoking. We give him job, incentive and salary. It’s work!. He also get his friends involve in this job. They help community with pleasure and nowadays we have more than 10 young smokers who can quit smoking so far”. *
Colorectal cancer	Method of colorectal cancer prevention was decrease eating food or vegetable contaminated with carcinogen and do screening with FIT. Decrease eating carcinogenic food was focused on decrease eating red meat and process meat. Some accredited health care professionals said *“We set up the project “Clean food Good Test” by educate community people to be aware of risk from red meat, grilled meat or carcinogenic food and encourage people to eat organic vegetable”*. For colorectal screening, one accredited health care professionals said *“We participated in the project using Fecal Immunochemical Test (FIT) in community people aged 45-74 Years old”. *
Cervical cancer	Cervical cancer prevention was implementation of HPV vaccination with female students who study in primary school, the age of 9-10 years. Including, provide education for adolescents to prevent sexual risk behaviors: decrease early sexual initiation and unsafe sex in adolescents. In addition, increasing of pap smear test for community women. Some accredited health care professionals said *“Before we do implementation we do situational analysis and find out their problems and need to participate in CA cervix screening. Women don’t like to do Pap smear. One accredited health care professional said “We have projects on mom girl prevention, unwanted pregnancy and unsafe sex. We promote parent-adolescent sexual risk communication. Adolescent also set up adolescent self-help group to help each other”*.
Breast cancer	Breast cancer prevention was focused on breast self-examination. Some accredited health care professionals said *“We have to encourage female to do breast self-examination at least 80% of national health indicators”. In addition, we need to prevent from Persistent Organic Pollutants (POPs) and promote physical activity”. *Moreover, tertiary prevention was done with people with breast cancer who has wound infection on her breast. Effective wound dressing and nutrition were implemented to prevent severe infection. One accredited health professional said *“After I provide wound care and encourage the patient to have proper food for wound healing. The wound was healed. She is now very happy”. *

## Discussion

Study showed some success of behavioral changes of 3E 2S such as smoking reduction and less eating raw fish. This is the evidence of life style modification contributes to decrease risk factors and behavioral changing which is congruence with the success of decreasing smoking behaviors of community people reduced risk factors for lung cancer (Gemine, et al, 2019). However, previous study showed there was no association between a positive FIT and meat consumption or other lifestyle (Pramual, 2018). 

Focusing on situational analysis by exploring community’s problems, need and ways to solve the problem from community people’s viewpoints is crucial before implementation. Accredited health professionals situational analysis by getting community people to participate in exploring community’s health problems, need and way to do cancer prevention before project implementation such as cancer screening. The implementation of programs needs specific emphasis on any obstacle to patient access (Waldman, 2013) and practice. Results from situational analysis help to design appropriate, practical, and sustainable to cancer prevention. 

Cancer screening and prompt treatment are essential strategies for secondary prevention. Mortality can be reduced if cases are detected and treated early (WHO, 2007). Study showed cancer prevention from the experiences of accredited health professional while working in the community include all primary, secondary and tertiary prevention of cancer. Not only cancer screening was done for secondary prevention but also prompt primary care was done for tertiary prevention as well. 

 Cancer prevention is not only based on specific knowledge and experienced skill of health care provider but include community people by getting them access to information, understanding, making decision, and empower them to have self- efficacy to change their risk behaviors. This is the way to improve health literacy for community people. Previous study showed enhancing knowledge and perceived self-efficacy could make concerning danger of eating raw fish and of developing cholangiocarcinoma (Laithavewat, 2018). 

In addition, study showed the crucial role of community health volunteer, health care provider partner, contributing to effective cancer prevention which congruence with important factors affecting behavioral changes were ability of community leaders and volunteers (Saranrittichai et al., 2012). To improve cancer prevention need to emphasis on capacity building of health care provider in terms of education, training and empowerment (Grimes, 2014) including health volunteers to take care of community people. Health care volunteer can act as home care workers. Home care worker who has trained skill can help health care provider work and enhance patients’ health behaviors (Smyth, 2015).

Study showed to improve cancer prevention, need to focus on quality of life of both community people and health care providers. Appropriate and effective health care service can promote people’s quality of life both for patients and family (Admi et al., 2013). Inspiration for working in community including cancer prevention need to focus on happiness of health care provider. 

It is suggested that community based approaches for cancer prevention need to set up for local policy to support health care provider in the community. Approaches need to focus on empowerment of health care provider, family and community. Integrated all level of preventions: primary, secondary and tertiary prevention need to be considered. Thus, health literate community need to be focus on community-based cancer prevention. 
